# Adverse Childhood Experiences Are Associated with Reduced Psychological Resilience in Youth: A Systematic Review and Meta-Analysis

**DOI:** 10.3390/children9010027

**Published:** 2021-12-31

**Authors:** Cyleen A. Morgan, Yun-Hsuan Chang, Olivia Choy, Meng-Che Tsai, Shulan Hsieh

**Affiliations:** 1 Institute of Creative Industries Design, National Cheng Kung University, Tainan 701, Taiwan; cyleenmorgan99@gmail.com; 2 Institute of Genomics and Bioinformatics, College of Life Sciences, National Chung Hsing University, Taichung 402, Taiwan; changyh@nchu.edu.tw; 3 Department of Psychology, Nanyang Technological University, Nanyang Avenue 48, Singapore 639818, Singapore; oliviachoy@ntu.edu.sg; 4 Department of Pediatrics, National Cheng Kung University Hospital, College of Medicine, National Cheng Kung University, Tainan 701, Taiwan; 5 Cognitive Electrophysiology Laboratory: Control, Aging, Sleep, and Emotion (CASE), Department of Psychology, National Cheng Kung University, Tainan 701, Taiwan; psyhsl@mail.ncku.edu.tw; 6 Institute of Allied Health Sciences, College of Medicine, National Cheng Kung University, Tainan 701, Taiwan; 7 Department of Public Health, College of Medicine, National Cheng Kung University, Tainan 701, Taiwan

**Keywords:** adverse childhood experience, resilience, adolescent, young adults, meta-analysis

## Abstract

Background: Adverse childhood experiences (ACEs) are presumed to influence internalizing and externalizing behaviors that can significantly debilitate long-term biopsychological development in individuals. Psychological resilience has been shown to effectively mediate the relationship between ACEs and negative health outcomes since individuals with low levels of resilience may have difficulty with bouncing back from toxic exposure to ACEs. Thus, the present systematic review and meta-analysis was aimed toward synthesizing current knowledge of the relationship between ACEs and psychological resilience in youths. Methods: A combination of key words relevant to the present study was searched on the PubMed, EMBASE, Scopus, Cochrane, and Google Scholar databases. The results were restricted to English publications and human studies, with subjects ranging between the age of 0 to 35 years. Effect-size measures inclusive of pooled correlation coefficients for correlation analyses and pooled odds ratios for regression analyses, respectively, were calculated using random-effect models to determine the relationship between ACEs and psychological resilience. Results: The searches identified 85 potentially relevant studies. Among them, 76 were excluded due to limited access, irrelevant data, and the fact that the variables of interest were not explicitly measured or disclosed, leaving a final total of nine studies considered valid for the meta-analysis. Findings from correlational meta-analysis (*n* = 6) revealed a significantly negative association between ACEs and resilience (β = −0.120 [−0.196, −0.043]). The meta-analysis of the studies (*n* = 3) reporting dichotomous outcomes (ACE ≥ 1 vs. no ACE) indicated that subjects who experienced an ACE were 63% less likely to display high resilience, in comparison to subjects without such experiences. Conclusion: Our results support a negative association between ACEs and psychological resilience and highlight the multiple dimensions that constitute resilience in an ACE-exposure context. These findings may be particularly useful to policy makers and healthcare institutions in terms of helping them devise effective medical interventions and community outreach programs intended to develop resilience in youths, thus reducing health-risk behaviors and negative health outcomes.

## 1. Introduction

Adverse childhood experiences (ACEs) have become a mounting concern among federal public health institutions due to their deleterious effects on the long-term trajectory of human development [1,2]. ACE refers to traumatic events experienced during childhood, such as violence (physical, sexual, and or emotional), parental neglect, or living with an adult experiencing a mental illness or engaging in substance abuse [3]. According to the United Nations Children’s Fund, over 40 million children have been subjected to one or more types of ACE, resulting in an increased risk of physiological disorders [2].

The current literature has identified a significant association between ACEs and intractable health issues, such as alcoholism, drug abuse, depression, suicide, poor physical health, and obesity [2,4,5,6,7]. This negative association is attributable to the constant activation of alarmingly high stress hormone levels in children, contributing to physical and mental maldevelopment [3,8]. Research has further found a positive linear relationship between ACE prevalence and adverse health outcomes. This finding is supported by Wang et al., who demonstrated that adults experiencing more ACEs are at a higher risk for negative physical and mental health outcomes. Despite a substantial amount of literature on this topic, there has been a paucity of studies focusing specifically on youth outcomes [9].

Hochberg et al. identified neurophysiological changes distinctive to youthhood [10]. This stage is characterized by a period of striking somatic and behavioral changes, which make way for susceptibility to substance abuse and other risky behaviors adopted as coping mechanisms [6,11]. According to the 2018 World Drug Report (published by the United Nations Office on Drugs and Crime), 18–25 year-olds are identified as the highest-risk group for drug use [12]. This highlights the ever-growing necessity to understand the bio-behavioral pathways linked to early risk exposure in order to mitigate negative consequences resulting from ACEs and improve later health and functioning in young adulthood [1,9,13].

A key characteristic of youths who are successful in combating ACEs is resilience. Resilience has been broadly defined as a mental process of negotiating, managing, and adapting to significant sources of stress or trauma [14]. Assets that facilitate positive adaptation to trauma are largely attributed to the presence of protective factors. Current research has identified three protective orientations of resilience: trait, outcome, and process. Trait resilience suggests that resilience is a stable personality trait (i.e., ego resilience and psychological hardiness) that intrinsically enhances individual adaptation to stress or adversity [15]. Outcome-oriented resilience is defined as a behavioral outcome that enables subjects to conquer and recover from substantial exposure to adversity [16]. Within this sphere, resilience outcome is regarded as modifiable and is considered to be partially determined by internal (i.e., epi/genetics, personality traits, and beliefs) and/or external (i.e., family, community support, and environmental resources) factors [2,3]. Finally, the process-oriented approach views resilience as a dynamic process of adaptation that enables individuals to actively adapt throughout periods of adversity [16].

Although the extant literature highlights the detrimental effects of ACEs on health outcomes, resilience is often considered to be an important mediator in this process [4,7,17,18,19]. Research suggests that resilience significantly mediates the impacts of ACEs on negative outcomes. That is, there is a seemingly causal decline in resilience along with ACE exposure [5,8,19,20]. Richter et al. (2019) further identified a significant association between ACE exposure and impaired functional neural connectivity, resulting in low resilience outcomes in children and young adults [8]. Thus, we hypothesize that increased ACE exposure is generally associated with lower levels of resilience at the early stage of the life course.

According to the rationale mentioned above, this paper is aimed toward synthesizing current evidence on the association between ACEs and psychological resilience among children, adolescents, and young adults. These cohorts are fundamentally distinct due to profound bio-psychological changes that are experienced in adolescents and emerging young adults [11]. Additionally, we anticipate that understanding the intricate linkage among the factors of interest can assist healthcare professionals with planning timely and effective interventions that will reduce the prevalence of risky behaviors among youth, thus facilitating a smooth transition into adulthood [1,4].

## 2. Materials and Methods

### 2.1. Search Strategy

A combination of key words such as “adverse childhood experience/s” OR “childhood adversity” AND “psychological resilience” OR “resilience” was searched on PubMed, EMBASE, Scopus, the Cochrane database, and Google Scholar.

### 2.2. Inclusion and Exclusion Criteria

The study selection process was based on assessing each article’s title, abstract, and full text. We limited the search to the period from 1985 up to 30 September 2021, specifically focusing on articles examining the relationship between ACEs and resilience in human subjects. Studies were excluded if resilience was treated as a moderating variable, and no statistical information was available on the factor’s direct relationship with ACEs, except in one instance where the author was contacted for disclosure of additional data. Articles were further excluded based on full-text accessibility. The cohort of interest was ideally individuals to 35 years of age. Therefore, studies on older participants were excluded from further analysis. The exclusion decision was made by the initial review author and independently assessed by a senior review author. The entire search process was performed following the Preferred Reporting Items for Systematic Reviews and Meta-Analyses (PRISMA) guidelines and registration.

### 2.3. Data Analysis

Microsoft Office Excel was used to organize articles for the systematic analysis by extracting information such as the title, year, mean age, ACE measure, resilience scale, and correlation coefficients between ACEs and resilience. Relevant data were subsequently imported into the Comprehensive Meta-Analysis Software version 3.0 for the meta-analysis.

Two meta-analyses were conducted to synthesize the effect sizes of the correlation coefficients in the correlation analyses and the odds ratios (ORs) in the regression analyses, as specified in the selected journal articles. When meta-analyzing the regression analyses, we dichotomized exposure to ACEs into two groups (i.e., ACE ≥ 1 vs. no ACE) and calculated the pooled OR of being highly psychologically resilient, as defined in the original papers, given that there were different questionnaires applied to measure resilience. The correlation coefficient and odds ratio at 95% confidence intervals (CIs) were calculated using a random-effects model. Results were considered statistically significant at a Z-test < 0.05, with a *p*-value < 0.01. The heterogeneity index was assessed using the I^2^ and Tau^2^ statistics and Q-value with its corresponding degrees of freedom.

## 3. Results

**Figure 1:**
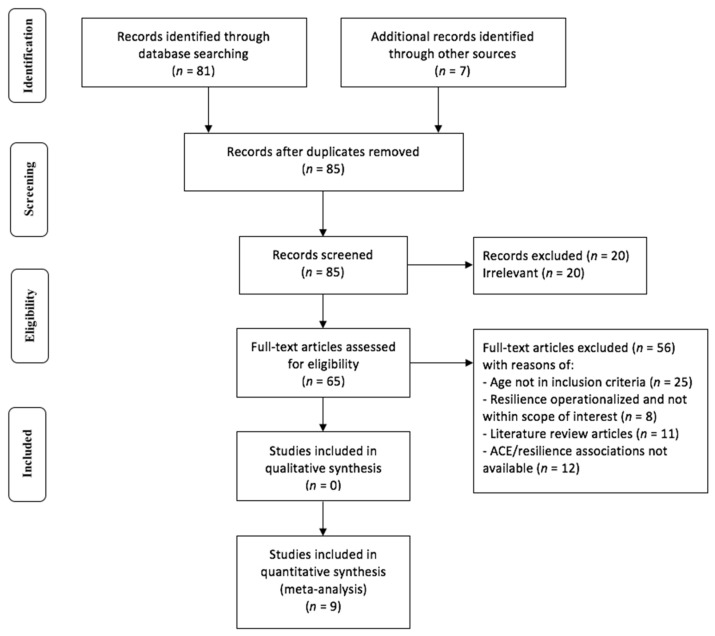
PRISMA flow chart illustrating search strategy.

Details of the selection process are specified in the PRISMA diagram (Figure 1) below. A total of 85 non-duplicate records were identified from the database searches. After screening, 20 articles were excluded due to irrelevance. Among the remaining 65 articles, 56 were further excluded because they were literature review articles, had insufficient statistical information (non-disclosure of the ACE-resilience association), and/or had variables (i.e., age, operationalized definition of resilience) that were not within the scope of the inclusion criteria. Characteristics of the nine remaining articles that qualified for the meta-analysis are specified in Table 1 below.

### Meta-Analysis Results

**Figure 2:**
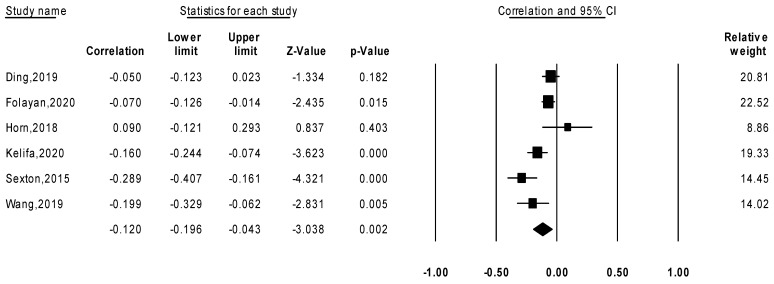
Random effect model for the meta-analysis of the correlation between adverse childhood experiences and psychological resilience.

A total of nine studies (161,165 participants) were in included in this systematic review. Among them, six independent studies (*n* = 2933) were used to conduct a correlational meta-analysis to examine the relationship between ACEs and psychological resilience. The pooled correlation coefficient was −0.120 (95% CI [−0.196, −0.043], *p* < 0.001) (Figure 2). The funnel plot was symmetrical (Figure S1), and the heterogeneity test revealed an I^2^ = 72.4% and a Tau^2^ = 0.006. The Eggers’ regression test determining the pooled correlation effects yielded an intercept = −1.458 (95% CI [−7.274, 4.358], *p* = 0.52), indicating a low likelihood of publication bias.

**Figure 3:**
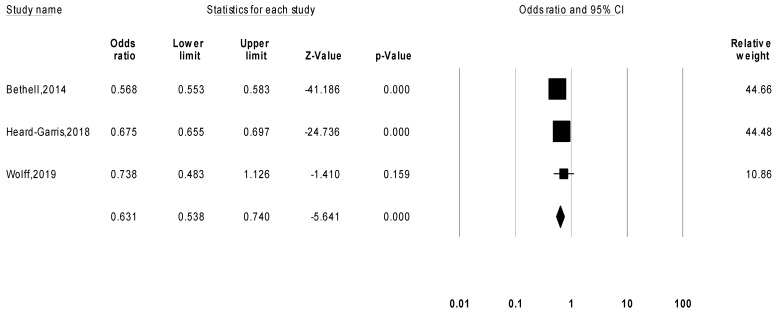
Random effects model for meta-analysis of the likelihood of subjects with ACEs being highly psychologically resilient as compared to those without adverse childhood experiences.

Another three studies (*n* = 158,232) that reported the likelihood of subjects who had been exposed to ACEs being psychologically resilient as compared to those who had not were meta-analyzed in a separate meta-analysis. The pooled odds ratio was 0.631 (95% CI [0.538, 0.740], *p* < 0.001) (Figure 3), indicating that subjects with ACEs are less likely to be psychological resilient. The funnel plot was asymmetrical (Figure S2), and the heterogeneity test revealed an I^2^ = 97% and a Tau^2^ = 0.015. Egger’s regression test for pooled odds ratio yielded an intercept = 1.871 (95% CI [−108.579, 112.322], *p* = 0.87), indicating a possible publication bias.

## 4. Discussion

The present review summarizes current knowledge of the relationship between ACEs and psychological resilience in youths. To our knowledge, this is one of the first meta-analytic efforts intended to comprehensively synthesize the relationship among the abovementioned variables focusing specifically on youths. Through a systematic review and meta-analysis, we identified a negative association between ACEs and psychological resilience. In other words, levels of resilience were lower among individuals with a greater prevalence of ACEs.

Central to this discipline, elucidating the various orientations of resilience is critical to ascertain associations or distinctions between co-existing protective factors [16,21,22]. Among the nine studies, three operationalized resilience using a process-oriented approach, referring to the ‘dynamic’ process of ‘actively’ coping with adversity, without physical and emotional dysfunctions [4,5,23]. Another three studies conceptualized resilience as outcome-oriented, where it was defined as the ability to ‘rebound’ or ‘bounce back’ from significant challenges [6,20,24]. In two other studies, outcome and process-oriented definitions overlapped, where resilience was defined as the ability to ‘cope’ and ‘recover’ from hardship while ‘adapting to change’ [19,25]. A single study adopted a trait-oriented approach and defined resilience as an individual’s ‘executive functioning’ capacity, comprising ‘inhibitory control’, ‘working memory’, and ‘mental flexibility’ [26]. Horn et al. suggests that each of these subdomains is predictive of adaptive functioning due to controlling cognitive receptors that support goal-directed behavior [26]. Taken together, these definitions highlight the intricacy of resilience, and suggest the critical need to understand the heterogenic constructs that contribute to resilience in individuals.

Aligned with most of current literature [5,16,19,20], findings from the correlational meta-analysis indicated a significant negative association between ACE exposure and psychological resilience. It was noted that all six studies that were analyzed yielded negative coefficients, with the exception of Horn’s (2018) [26] study, which examined the relationship between poly-victimization and executive functioning among children living in foster care. The results revealed a positive but insignificant association. However, scores in executive functioning were significantly lower in children under foster care than in children from the community [26]. While several explanations may underlie this discrepancy (i.e., limited subdomain measures from neuropsychological testing and a small sample size), genetic effects on the behavioral dimensions of resilience should not be underestimated [18,27,28]. Wolf’s (2018) classical twin study investigating the relationship between post-traumatic stress disorder and psychological resilience revealed a negative correlation, attributing 59% of its correlate to the presence of a single genetic factor [29]. Similarly, Niitsu (2018) identified the L/L and L’/L’ and S/S and S’/S’ genotype of the 5-HTTLPR (rs25531) genetic variant as significant contributors to psychological resiliency among children, adolescents, and adults, respectively [28]. Additional studies have also identified epigenetic changes in terms of an increased methylation of a glucocorticoid receptor promoter in the hippocampus, which functions as a stress regulator in individuals who are exposed to prolonged adversity [18,27,28]. Taken together, these findings show inextricable evidence of the role of genetics and heritability on resilience. Trait resilience in particular facilitates the development of bio-behavioral protective factors, which enable stressful coping in conditions of adversity [16,17,30]. This likely explains why a slightly positive association was identified between poly-victimization and emotional functioning among foster children [26].

The meta-analysis of the studies reporting dichotomous outcomes confirmed that the subjects were 63% less likely to manifest psychological resilience against ACEs. Among the three meta-analyzed studies, two studies obtained data from the National Survey of Children’s Health, which likely explains the similar resilience outcomes [4,20]. A greater discrepancy was observed in Wolff’s (2019) study, which investigated the relationships between ACEs, resilience, and mental and behavioral health conditions in pregnant women [19]. Largely because of age discrepancy in the observed subjects and study contexts, there was no significant difference found between pregnant women with low and high resilience on the mean number of ACEs. In addition, all papers that examined the role of family and community presence as external protective components further confirmed a significant association between these factors and increased psychological resilience. Significant covariates included family support, attending religious activities, eating and sharing ideas with one’s family, safe, clean neighborhoods with communal amenities, and mentorship from community members [4,6,20,23].

Based on the current systematic review, we tentatively concluded that the promotion and sustenance of resilience occur as a result of the dynamic interplay of genetic and environmental influences [31,32]. Liu et al. (2018) characterized the interaction of these mechanisms as epigenetics [33]. Epigenetics play a critical role in altering phenotypic and behavioral outcomes by reprogramming gene expression in response to changes in lifestyle trajectories [26,34]. This analysis strengthens our understanding of resilience as a complex, adaptive system of interacting genetic and environmental factors aimed to improve individual problem-solving and coping capacities [26,33,34]. Concurring with the current literature, these findings highlight the critical need for community leaders and policy makers to gain a systems-oriented understanding of psychological resilience in order to devise adequate resilience-promoting community programs to help strengthen external protective factors among individuals with ACE exposures [16,35].

Although the present meta-analysis was conducted in accordance with standardized practices, some limitations must be addressed. Firstly, there were large discrepancies in the ACE and resilience scales implemented in each study. This reflects the complexity of the measured dimensions of ACE and resilience, which may be attributed to less than optimal standardization when meta-analyzing results. Secondly, the present study only assessed psychological resilience as a unidimensional variable. Moreover, the extant literature has heavily regarded resilience as a mediating variable [4,14,25,26]. Future research should incorporate more flexible operational definitions for resilience so as to examine its different impacts on adverse biopsychological health outcomes. Thirdly, age could be a significant moderator of the relationship between ACEs and resilience. The present meta-analysis was limited to a young population, so caution must be taken when generalizing such findings to an older age group.

## 5. Conclusions

The present meta-analysis investigated the relationship between ACEs and resilience among youths. Findings from the meta-analysis showed a significant negative association between both variables. Given the heterogeneity among the included studies, there is an urgent necessity to comprehensively understand the multifaceted orientations that constitute resilience in an ACE-exposed context. As contemporary understanding of resilience has shifted toward systems theory [33], the complex task of further elucidating what promotive and protective factors as well as risks and vulnerabilities in response to early adversities are related to resilience outcomes, which was out of the scope of the present meta-analysis, remains an important area for future research. These findings are expected to be critical to health-care institutions and policy makers in terms of their devising effective intervention measures to develop resiliency among youths in order to curtail the prevalence of risky behavior and improve lifelong health.

## Figures and Tables

**Table 1:** Characteristics of the included studies.

Study	Population	Sample Size	Age	Name of ACE Measure	ACEs Measured	Resilience Scale	Association with Resilience	Covariates Examined
Heard-Garris et al. (2018)	National Survey of Children’s Health; United States population	62,200	0–17	NSCH-ACEs ^1^	9	Parent-perceived resilience scale	Negative	Eating meals together *
								Religious attendance *
								Sharing ideas with children *
								Neighborhood amenities and mentorship *
Wolff et al. (2019)	Pregnant women (~14–23 weeks of gestation); United States population	355	22–38	BRFSMQ ^2^	8	CD-RISC 10 ^7^	Negative	None
Bethell et al. (2014)	National Survey of Children’s Health; United States population	95,677	0–17	NSCH-ACEs	9	“Staying calm and in control when faced with a challenge”	Negative	Protective home environment
								Healthy parents
								Supportive community
Ding et al. (2019)	Gay/bisexual men; Chinese population	714	19–35	Kaiser-CDC study	10	CD-RISC10	Negative	None
Folayan et al. (2020)	Nigerian population	1209	11–16	ACE questionnaire ^3^	10	CD-RISC10	Negative	Social support
Kelifa et al. (2020)	Eritrean college students	507	18–25	ACE-IQ ^4^	13	CD-RISC10	Negative	None
Sexton et al. (2015)	4-months post-partum mothers	214	23–33	CTQ ^5^	28	CD-RISC10	Negative	None
Horn et al. (2018)	88 foster-care and community children	88	3–4	MCS ^6^	5	NEPSY ^8^	Positive	None
Wang et al. (2019)	Taiwanese youth population	200	15–22	ACE-IQ	14	Inventory of Adolescent Resilience (Chinese version)	Negative	Household financial status
								Parental education
								Family support *

^1^ National Survey of Children’s Health- Adverse Childhood Experiences; ^2^ Behavioral Risk Factor Surveillance System Questionnaire; ^3^ Adverse Childhood Experiences Questionnaire; ^4^ Adverse Childhood Experiences International Questionnaire; ^5^ Childhood Trauma Questionnaire; ^6^ The Maltreatment Classification System; ^7^ Connor–Davidson resilience scale; ^8^ A Developmental NEuroPSYchological Assessment; * indicates significance (*p* < 0.05).

## Data Availability

The data presented in this study are available upon request of the respective author.

## Supplementary Materials

The following are available online at https://www.mdpi.com/article/10.3390/children9010027/s1. Figure S1: Funnel plot showing low publication bias for meta-analysis of the correlation between ACEs and resilience. Figure S2: Funnel plot showing high publication bias in the meta-analysis of the association between ACEs and resilience using dichotomous outcomes.
