# Supplementary material for: Adverse Childhood Experiences Are Associated with Reduced Psychological Resilience in Youth: A Systematic Review and Meta-Analysis
Source: Children (Basel). 2021 Dec 31;9(1):27. doi: 10.3390/children9010027 (PMC8773896; doi:10.3390/children9010027)

Figure S1. Funnel plot showing low publication bias for meta-analysis of the correlation between ACE and resilience.

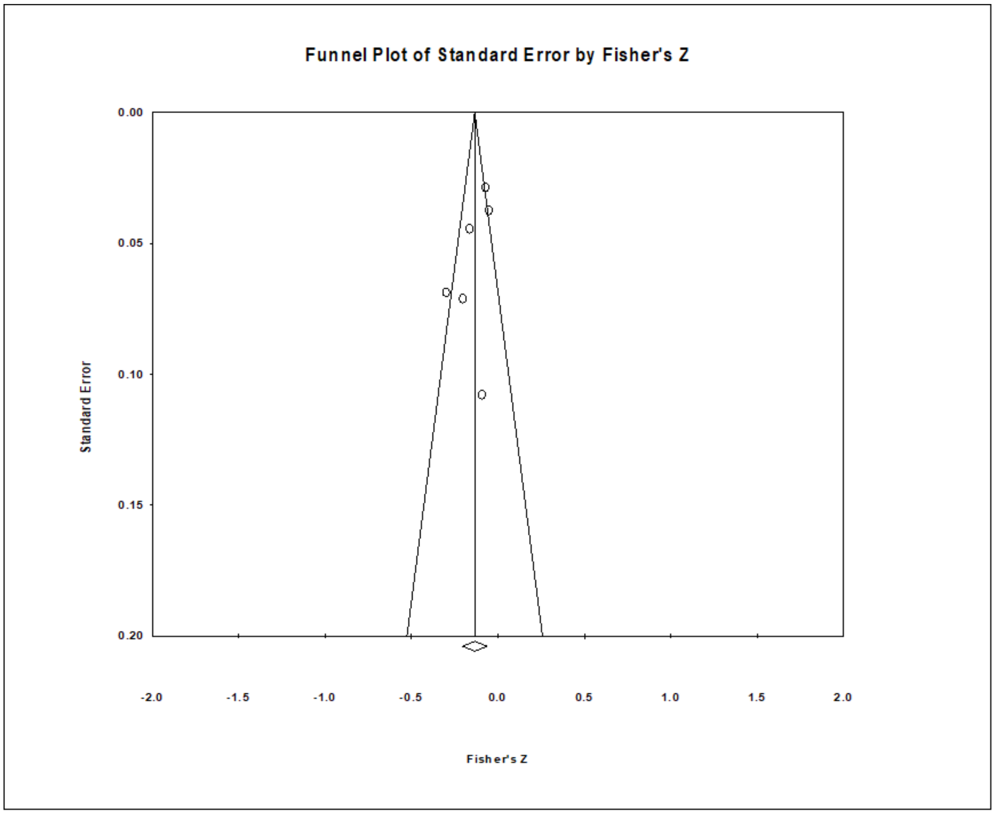

Figure S2. Funnel plot showing high publication bias for meta-analysis of the association between ACE and resilience using dichotomous outcomes.

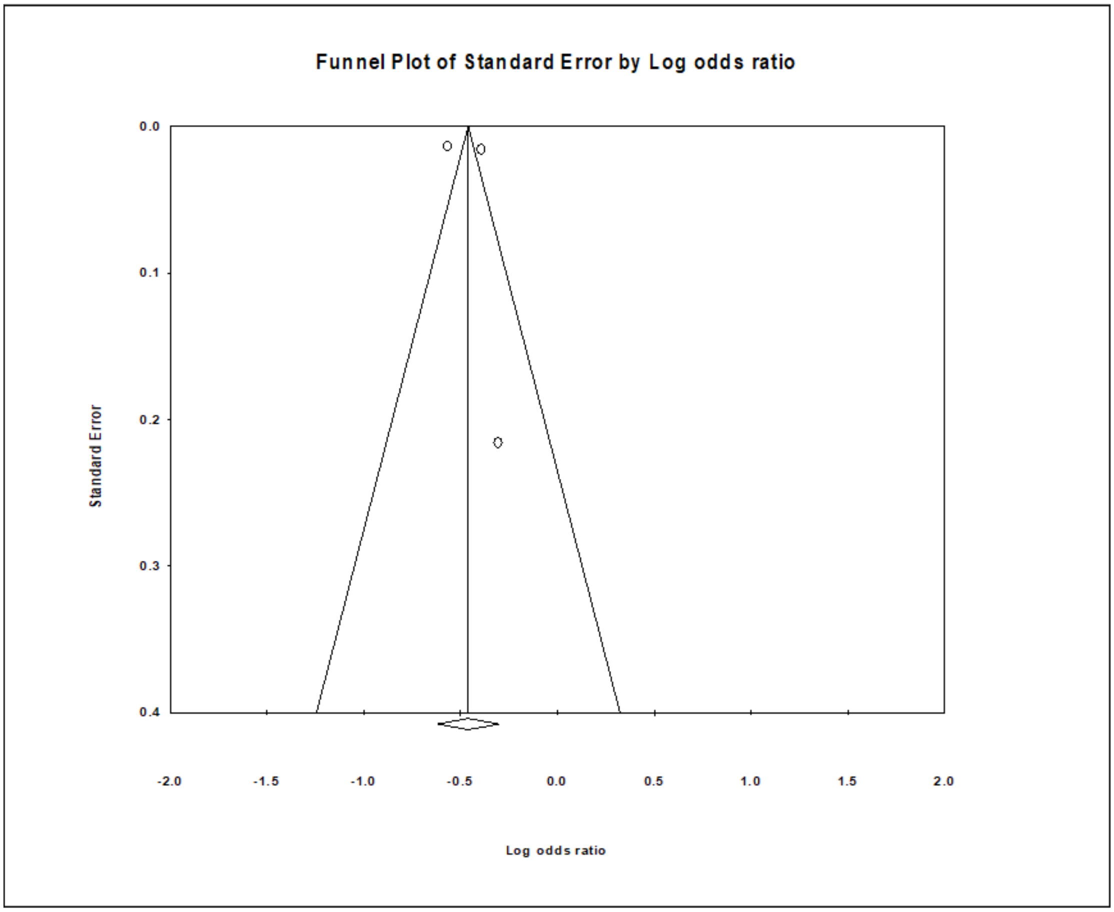

Supplement: Supplementary file 1 [file children-09-00027-s001.zip › children-1488510-supplementary.pdf]
